# The Complex Intersection of Race and Rurality: The Detrimental Effects of Race-Neutral Rural Health Policies

**DOI:** 10.1089/heq.2021.0136

**Published:** 2022-04-28

**Authors:** Stacy Grundy, Beth Prusaczyk

**Affiliations:** ^1^ Department of Population Science and Policy, Center for Rural Health and Social Service Development, Southern Illinois University School of Medicine, Springfield, Illinois, USA.; ^2^ Division of General Medical Sciences, Department of Medicine, Institute for Informatics, Washington University School of Medicine in St. Louis, St. Louis, Missouri, USA.

**Keywords:** race, rural, health policy, disparities

## Abstract

In this commentary, we discuss our experiences as women of different races growing up in the same rural area and how these experiences relate to health and health policy. Despite nearly five million Black people living in nonmetro areas, rural Black Americans face erasure in the rural narrative and the policies enacted to support them. This is detrimental to the overall uplifting of rural communities and to the elimination of the compounded disparities of being rural and Black. We aim to bring to life rural America for Black and White residents and the impact of the policies that shape it.

The narrative about rural America often centers on the “White working-class,” despite 25% of the rural population being non-White (with higher and lower percentages depending on the region).^1^ This narrative also often highlights the close-knit communities found in rural America. Indeed, compared with urban dwellers, rural Americans are more likely to see their communities as safe, friendly, and close knit, and share similar socioeconomic experiences.^2,3^ This creates a sense of solidarity for rural Americans that crosses racial and even state lines. As two rural health researchers—one of us White and one of us Black—we immediately bonded when we learned that we were both from the same rural area.

Even though we did not know each other growing up, after discussing our upbringings, we were struck by the similarities, including times when we saw our communities rally to support us. In two separate incidents, years and miles apart, we each experienced the natural disaster of flooding. Our homes and many of our personal belongings were destroyed or damaged and our families were faced with rebuilding after the trauma. B.P. remembers her neighbors bringing their johnboat to her front door to help transport her family's belongings out of the house in the chest-deep water.

S.G. remembers members of a neighboring community's church, which was predominately White, assisting her family as they removed water-damaged carpet and furniture from their home. When asked what it was like growing up in a rural area, we both cite memories such as these as examples of the benefits of living in small communities.

However, this similar experience also serves as an example of the differences between rural White and rural non-White Americans. B.P.'s home flooded suddenly when her town's aging wastewater treatment plant failed, causing flashflood waters from a recent storm to back up and flood the town. S.G.'s home, located in a town near the confluence of the Mississippi and Ohio rivers, flooded when the rivers overflowed due to a particularly wet spring. However, this weather event was made significantly worse due to overt racism among local residents, elected officials, and the federal government when delaying the decision to divert flood waters away from the historically Black town of Cairo, Illinois, and onto local farmland.

When a local U.S. congressman was asked whether he would rather have the farmland flooded or Cairo, he replied without hesitation, “Cairo. I've been there. Trust me…Have you been to Cairo? Ok, then you know what I'm saying then.”^4^ The decision to delay caused significant and avoidable damage to the town.^5^

It can be said that Black and White rural residents live in two different worlds. Although the above example of flooding relates primarily to rural infrastructure, agriculture, economics, and politics, as we discuss hereunder, these constructs cannot be separated from health and health policy. In this commentary, from our unique perspectives, we outline how health policies that target rural areas (place-intentional policies) but that remain neutral on race actually further racial disparities in rural areas and have a detrimental impact on the health of all rural residents. We argue that to improve the health of all rural residents, and particularly for non-White rural residents, we must enact policies that are not only place intentional but also *race intentional* for rural areas.

Place-neutral health policies are those that fail to consider their differential impact on rural versus urban areas, despite knowing the multitude of ways in which these areas differ.^6^ One example of this type of policy is Accountable Care Organizations (ACOs), which are managed-care models aimed at improving health and continuity of care among Medicare patients while also reducing overall health care costs. Rural residents generally have worse health, higher rates of poverty, and more barriers to care than urban residents,^7^ which suggests they could greatly benefit from ACOs.

However, it is especially challenging for rural health care providers to transform into ACOs due to the significant financial and infrastructure investments necessary to make the transformation. This policy fails to consider the differences between rural and urban areas, which diminishes its value at improving rural health despite there being an increased need in rural areas.

Policies that are instead tailored to rural areas (place-intentional policies) not only help to avoid the unintended consequences of place-neutral policies but also make possible significant improvements unique to rural life.^6^ We believe that just as we need place-intentional policies for rural areas, we also need *race-intentional policies* for rural areas. Place-intentional and *race-intentional* policies would consider not only the differences between urban and rural areas but also the differences between non-White and White rural residents.

Health disparities of rural residents have been an area of interest for many notable public health institutions, but despite being the second largest rural minority, the plight of rural Black Americans has ultimately been hidden.^8^ Although rural communities share a common multitude of health challenges,^9^ those challenges vary in severity based on race and ethnicity.^10^ According to a study conducted by the American Heart Association, Black rural residents are more than two to three times more likely to die from diabetes and high blood pressure than White rural residents,^11^ despite the rate of deaths related to diabetes and high blood improving among Black urban residents.^11^

Furthermore, non-White rural residents are less likely to have access to health care compared with White rural residents.^12^ The reasons for these disparities include both institutional racism, which creates structural barriers to health care access and utilization among rural non-White residents, and personally mediated racism perpetrated by rural health care providers on their non-White patients.^13,14^

Environmental racism is also a factor, which is not only used to harm non-White communities but is also instrumental in the building up of White communities.^15^ This is all within the historical context of medical racism perpetrated on the non-White community, in particular the Black community, through atrocities such as the Tuskegee Experiment^16^ and the forced sterilization of non-White women, particularly Black women, in the south.^17^

Furthermore, health and health care cannot be separated from other aspects of rural life. For example, in rural counties, racial disparities in unemployment were associated with higher rates of fair/poor health among Black residents,^18^ and rural Black residents were more likely to report not having accessed health care due to cost.^12^ Given the higher rates of unemployment, lower average per capita income, and worse health outcomes among all rural residents, these compounding racial disparities are particularly concerning. This is why policies created intentionally for rural communities that remain neutral in terms of race hinder the health benefits of those policies.

Rural hospital closures are a prime example of where place-intentional *and race-intentional* policies are needed. All of rural America is concerned about its hospitals closing and each community is vying for the limited support available to help keep its hospital open. However, like nearly everything in the United States, rural hospital closures have had a disproportionate impact on non-White rural communities. Thomas, Holmes, and Pink analyzed rural hospital closures over a decade and found that “when a rural hospital closes, it is likely serving a community with a relatively high percentage of Blacks and Hispanics” (p. 200).

The authors suggest one reason for this might be that rural communities with higher average per capita income were better able to retain their rural hospitals and that rural communities with relatively high percentages of non-White residents had lower average per capita income, which means they were more vulnerable to hospital closures. Another factor that could be contributing to this is racial segregation, both within the hospitals themselves and in the rural communities. Others have posited that the “residue” of a rural hospital previously only serving Black and non-White patients due to segregation may affect that hospital's reputation and the care delivered there today.^19^

And residential segregation has been long linked to worse health and health care for non-Whites.^20^ This highlights the complex intersection of race, the economy, housing, and health in rural America. Indeed, our own experiences remind us of this disparity. B.P. is from a county that is 97.7% White and 0.3% Black^21^ and has one hospital. S.G. is from a county that is 60.9% White and 35.4% Black^22^ and, strikingly, it has not had a hospital since 1986.

An assessment (conducted by the authors, as no research exists assessing federal policies aimed at preventing rural hospital closures for their specific focus on race) of the 12 existing federal policies aimed at supporting rural hospitals and preventing additional closures^23^ reveals that none are intentional in (or even nominally identify) their efforts to prevent closures specifically among rural non-White communities, despite this compounded disparity. The Delta Region Community Health Systems Development Program^24^ is the closest existing rural hospital closure policy to be race intentional but only in that it is focused on the Mississippi Delta region, which has a larger proportion of Black and African American rural residents than other rural areas in the United States.^1^

An example of a *race-intentional* health policy, though not *place intentional,* is the Center for Disease Control and Prevention's Racial and Ethnic Approaches to Community Health (REACH 2010) project. REACH sought to eliminate racial and ethnic disparities in six key health priority areas (e.g., infant mortality and diabetes) by supporting the community coalitions in the design, implementation, and evaluation of community-driven strategies to eliminate health disparities, and in many communities, disparities were reduced.^25^

One could imagine a similar policy for eliminating rural health disparities, but, we argue, without that policy considering the differential impact it may have on White and non-White rural residents, the policy will fail to improve overall rural health disparities as well as the racial and ethnic health disparities among all rural residents.

The erasure of non-White Americans from the rural narrative means they have seen fewer improvements in their communities and the advancement of rural communities as a whole has been stymied. It is our hope that by reinserting non-White residents into the rural narrative and enacting *race-intentional* rural health policies, we can begin to disentangle this intersectional and compounded bias in America. Furthermore, although we know a rising tide does not lift all ships, we strongly call for policies that will improve all aspects of rural life, such as the economy and infrastructure, for all rural residents.

This is because at the intersection of race and class in America is a White supremacist history of pitting working-class Whites against working-class non-Whites in an effort to keep the oppressed, oppressed, and the oppressors, the oppressors. We echo the calls from others for state and federal policies aimed at reversing this history, specifically as it relates to health,^26^ and bringing rural White and non-White residents together. And together, we can work to eliminate not only rural–urban disparities but also racial disparities in our communities.

## Abbreviations Used

ACOs: Accountable Care Organizations
REACH: Racial and Ethnic Approaches to Community Health
